# The Role of Metals and Trace Elements in the Pathogenesis of Osteoarthritis and Other Rheumatic Diseases

**DOI:** 10.3390/ijms27177889

**Published:** 2026-09-04

**Authors:** Elżbieta Krzemińska, Beata Tarnacka, Agnieszka Ścibior

**Affiliations:** 1 Faculty of Medicine, Collegium Medicum, Cardinal Stefan Wyszynski University, Dewajtis 5, 01-815 Warsaw, Poland; elzbietakrzeminska10@gmail.com; 2 Department of Rehabilitation, Faculty of Medicine, Medical University of Warsaw, 02-091 Warsaw, Poland; 3 National Institute of Geriatrics, Rheumatology and Rehabilitation, Spartańska 1, 02-637 Warszawa, Poland; 4 Department of Biomedicine and Environmental Research, Institute of Biological Sciences, Faculty of Medicine, The John Paul II Catholic University of Lublin, Konstantynów 1J, 20-708 Lublin, Poland; agnieszka.scibior@kul.pl

**Keywords:** osteoarthritis, rheumatic diseases, trace elements, metal homeostasis, oxidative stress, ferroptosis, metalomics, biomarkers

## Abstract

Osteoarthritis (OA) and other rheumatic diseases are among the leading causes of chronic pain, disability, and reduced quality of life worldwide. Increasing evidence indicates that disturbances in metal homeostasis contribute to the pathogenesis of these disorders through their effects on oxidative stress, immune regulation, cartilage metabolism, bone remodeling, and extracellular matrix degradation. This review summarizes current knowledge regarding the role of essential metals and trace elements, including zinc, iron, copper, magnesium, selenium, and manganese, as well as toxic metals such as cadmium, lead, mercury, and arsenic, with primary emphasis on their involvement in the development and progression of osteoarthritis. Other rheumatic diseases are discussed briefly to provide a comparative perspective. Particular attention is given to the molecular mechanisms underlying metal-mediated pathology, including oxidative stress, ferroptosis, mitochondrial dysfunction, activation of intracellular signaling pathways, and inflammasome activation. The diagnostic and therapeutic potential of metal-related biomarkers, metalomics, and targeted interventions aimed at restoring metal homeostasis are also discussed. Furthermore, current limitations of available studies and emerging research directions, including single-cell technologies, spatial transcriptomics, multi-omics approaches, and personalized medicine, are highlighted. A better understanding of metal homeostasis may improve the identification of novel biomarkers and therapeutic targets, ultimately contributing to more precise diagnosis and individualized treatment strategies for osteoarthritis and other rheumatic diseases.

## 1. Introduction

Rheumatic diseases constitute a major public health concern due to their high prevalence, chronic course, and substantial impact on physical function and patients’ quality of life [1,2]. The most important entities include osteoarthritis (OA), rheumatoid arthritis (RA), ankylosing spondylitis (AS), gout, and systemic lupus erythematosus (SLE). Among these, osteoarthritis is of particular clinical importance, as it is the most common joint disorder and one of the leading causes of chronic pain and disability in adults and older individuals [1,3].

The pathogenesis of rheumatic diseases is multifactorial and involves the interplay of inflammatory processes, immune dysregulation, oxidative stress, cartilage degradation, and bone remodeling [1,2,4]. Contemporary concepts of OA extend beyond the traditional view of the disease as a purely mechanical and degenerative disorder, emphasizing the active involvement of chondrocytes, synovial tissue, subchondral bone, and numerous inflammatory mediators in the development of structural joint damage [1,2]. Likewise, in RA, AS, gout, and SLE, chronic inflammation contributes to progressive tissue destruction, disruption of joint homeostasis, and perpetuation of the disease process [5,6,7].

Over the past decade, increasing evidence has suggested that disturbances in the homeostasis of metals and trace elements play a significant role in the pathogenesis of rheumatic diseases [3,5,8]. Trace elements such as zinc, copper, selenium, manganese, magnesium, and iron serve as essential cofactors for numerous enzymes involved in cellular metabolism, immune regulation, maintenance of redox balance, and extracellular matrix synthesis [3,8]. Alterations in their concentrations may influence the expression of pro-inflammatory cytokines, the activity of immune cells, cartilage and bone metabolism, and the progression of degenerative processes within the joint [3,5,8].

Particular attention has been given to the role of metals in the regulation of oxidative stress, which is recognized as one of the key mechanisms responsible for tissue damage in rheumatic diseases. Excessive production of reactive oxygen species (ROS) amplifies inflammatory responses, damages lipids, proteins, and DNA, and activates signaling pathways involved in cartilage degradation and bone remodeling [4,9]. Trace elements function as cofactors for major antioxidant enzymes, including superoxide dismutase, catalase, and glutathione peroxidase; therefore, both deficiency and excess of these elements may impair antioxidant defense mechanisms and influence disease progression [3,4,8].

The significance of metals in rheumatic diseases extends beyond their physiological functions. An increasing number of experimental and clinical studies have demonstrated that alterations in the concentrations of specific trace elements, particularly zinc and copper, correlate with inflammatory activity in rheumatoid arthritis and levels of pro-inflammatory cytokines [5,6,10]. At the same time, growing attention has been paid to the detrimental effects of toxic metals, including cadmium, lead, mercury, and arsenic, which may enhance oxidative stress, impair immune function, and promote chronic inflammation, thereby contributing to the development or exacerbation of rheumatic diseases [11,12].

Although the majority of available evidence concerns osteoarthritis, increasing data indicate that disturbances in metal homeostasis also play an important role in other inflammatory and autoimmune rheumatic diseases [5,6,7,8]. Mechanisms associated with oxidative stress, immune dysregulation, and abnormalities in cartilage and bone metabolism represent common pathogenic pathways shared by many of these disorders [4,8,9].

The primary aim of this review is to summarize current knowledge regarding the role of metals and trace elements in the pathogenesis of osteoarthritis, with particular emphasis on the molecular mechanisms linking metal dyshomeostasis to cartilage degeneration and disease progression. Other rheumatic diseases are discussed only briefly to provide a comparative perspective and to distinguish OA-specific mechanisms from broader alterations in metal and trace element homeostasis reported across rheumatic disorders. Particular attention is given to oxidative stress, regulated cell death, mitochondrial dysfunction, inflammatory signaling, bone remodeling, cartilage degradation, and the potential diagnostic and therapeutic implications of disturbed metal homeostasis.

## 2. Metal Homeostasis in the Human Body

Metal homeostasis is one of the fundamental mechanisms underlying normal physiological function, ensuring metabolic balance, tissue integrity, and the proper regulation of immune responses. Biologically active metals and trace elements, including iron, zinc, copper, manganese, and selenium, are of particular importance because they participate in numerous enzymatic, redox, and signaling processes. Their concentrations are tightly regulated at the levels of intestinal absorption, transport, storage, and excretion, and disturbances in these regulatory mechanisms may result in either functional deficiency or toxic accumulation of metals within tissues [13,14,15,16].

### 2.1. Regulatory Mechanisms

One of the key components of metal homeostasis is a network of membrane transporters responsible for the uptake, intracellular trafficking, and export of metal ions [13,14,16]. In iron metabolism, the transferrin receptor, ferroportin, and hepcidin—a peptide hormone recognized as the master regulator of systemic iron homeostasis—play central roles. Hepcidin binds to ferroportin, promoting its internalization and degradation, thereby limiting iron release from enterocytes, macrophages, and hepatocytes into the circulation [13,17]. Specialized transporters have also been identified for zinc and copper, regulating their intracellular concentrations in the cytoplasm and cellular organelles and thereby ensuring the proper activity of numerous enzymes, signaling pathways, and cell survival mechanisms [14,15]. Similar regulatory systems have been described for other biologically active metals, including manganese, whose intracellular homeostasis is maintained through the coordinated activity of specific membrane transporters [16].

Metallothioneins constitute another important protective component of metal homeostasis. These low-molecular-weight, cysteine-rich proteins exhibit a high affinity for zinc, copper, and cadmium ions [18]. They play essential roles in heavy metal detoxification, regulation of trace element bioavailability, and protection against oxidative stress. Furthermore, metallothioneins contribute to the regulation of inflammatory and immune responses by controlling the biological availability of metal ions and modulating the activity of zinc-dependent transcription factors [18,19].

Ferritin plays a pivotal role in iron homeostasis as the principal intracellular iron-storage protein, maintaining iron in a biologically safe form. Iron sequestration by ferritin limits the participation of free Fe^2+^ ions in the Fenton reaction, thereby reducing the generation of reactive oxygen species and protecting cells from oxidative damage [20,21]. In addition, ferritin is an acute-phase protein, and its circulating concentration may reflect both body iron stores and the intensity of inflammatory activity [17,20].

Iron transport in plasma is mediated primarily by transferrin, which binds ferric (Fe^3+^) ions and delivers them to cells through interaction with the transferrin receptor. This mechanism ensures tightly controlled iron utilization while minimizing its potential toxicity [13,22]. Ceruloplasmin, a copper-containing acute-phase protein with ferroxidase activity, also plays an important regulatory role. By oxidizing ferrous (Fe^2+^) to ferric (Fe^3+^) iron, ceruloplasmin facilitates iron binding to transferrin and thereby coordinates iron and copper metabolism, contributing to the maintenance of systemic metal homeostasis [22].

### 2.2. Disturbances of Metal Homeostasis During Chronic Inflammation

Chronic inflammation induces complex disturbances in metal homeostasis, affecting metal absorption, transport, storage, and redistribution between plasma and tissues [13,17,23]. One of the best-characterized mechanisms involves dysregulation of iron metabolism, in which pro-inflammatory cytokines—particularly interleukin-6 (IL-6)—increase hepcidin expression through activation of the Janus kinase/signal transducer and activator of transcription 3 (JAK/STAT3) signaling pathway [17,23]. This results in decreased circulating iron availability and increased iron sequestration within macrophages, representing a characteristic feature of both the acute-phase response and chronic inflammation [13,17,23].

Concomitantly, alterations in the concentrations of other essential metals, particularly zinc and copper, have been observed, with serum levels frequently correlating with inflammatory activity [15,24]. Patients with rheumatoid arthritis consistently exhibit decreased serum zinc concentrations accompanied by elevated copper levels [24]. These changes have been associated with activation of the acute-phase response, increased production of pro-inflammatory cytokines, and enhanced oxidative stress [15,24].

Disturbances in metal homeostasis also affect bone tissue, which serves both as a dynamic reservoir for numerous trace elements and as a site of their intensive utilization during bone remodeling. Chronic inflammation disrupts the balance between osteoblast and osteoclast activity, thereby affecting bone mineralization, bone resorption, and the local availability of metals essential for normal skeletal metabolism [25,26,27].

Significant alterations have also been reported in synovial fluid, which reflects the local biochemical environment within affected joints. Studies have demonstrated that concentrations of copper, iron, and zinc in the synovial fluid of patients with rheumatoid arthritis differ from those observed in serum, suggesting local redistribution of metals associated with inflammatory activity within diseased joints [28]. In osteoarthritis, alterations in metal concentrations within synovial fluid and joint tissues are increasingly recognized as potential contributors to disease pathogenesis and progression [25,26,29,30].

### 2.3. Summary

In summary, metal homeostasis relies on tightly regulated mechanisms governing metal transport, storage, and detoxification, in which membrane transporters, metallothioneins, ferritin, transferrin, ferroportin, hepcidin, and ceruloplasmin play central roles [13,14,15,16,17,18,19,20,21,22,23]. Disturbances of these regulatory pathways should not be regarded merely as consequences of chronic inflammation but rather as active contributors to the pathogenesis of rheumatic diseases through their effects on oxidative stress, immune responses, cartilage metabolism, and bone remodeling [15,18,19,24,25,26,28,29,30].

## 3. Essential Metals and Trace Elements in the Pathogenesis of Osteoarthritis

Essential metals play a pivotal role in maintaining the homeostasis of articular cartilage, subchondral bone, and the cells involved in the pathogenesis of osteoarthritis (OA) [3,8,31]. Increasing evidence indicates that disturbances in their homeostasis are not merely a consequence of disease progression but actively contribute to the initiation and advancement of degenerative joint changes by modulating inflammatory responses, oxidative stress, extracellular matrix metabolism, and chondrocyte survival [3,31,32]. These metals participate in numerous enzymatic reactions, extracellular matrix synthesis, and antioxidant defense mechanisms, whereas both deficiency and excessive accumulation may promote catabolic processes and accelerate OA progression [3,8,31].

It should be emphasized that circulating concentrations of trace elements are influenced by several factors, including age, sex, nutritional status, geographic region, biological matrix, and analytical methodology. Therefore, universal reference ranges cannot always be directly applied across studies, and comparisons between patients with OA and healthy controls should be interpreted in the context of the population and analytical method used [33,34]. Representative reference intervals for selected essential metals and trace elements are summarized in Table 1. These values should be interpreted as population- and method-dependent reference intervals rather than as disease-specific diagnostic thresholds.

Biological matrix represents an additional source of variability in trace element assessment. Paired analyses in patients with OA have demonstrated that elemental concentrations in synovial fluid are generally lower than those in the corresponding serum, with significant differences reported for Cu, Mg, and Zn; nevertheless, significant positive correlations between the two compartments have been observed for selected elements, including Cu and Mg [34]. Comparative analyses in patients with OA and RA have likewise demonstrated matrix- and disease-dependent differences in Se, Cu, Zn, and Fe concentrations between synovial fluid and plasma [28]. These findings indicate that circulating trace element concentrations cannot be directly extrapolated to the intra-articular environment and should be interpreted according to the biological matrix examined.

### 3.1. Zinc

Zinc is one of the most important trace elements involved in the pathogenesis of OA [3,31]. It regulates the activity of numerous enzymes responsible for extracellular matrix remodeling and degradation. Particular attention has been given to matrix metalloproteinases (MMPs), especially MMP-13, and a disintegrin and metalloproteinase with thrombospondin motifs 5 (ADAMTS-5), which mediate the degradation of type II collagen and aggrecan—the principal structural components of articular cartilage [31,32].

One of the best-characterized molecular mechanisms involves activation of the zinc transporter ZIP8 (SLC39A8) [31]. Increased Zn^2+^ influx into chondrocytes activates the ZIP8–metal-regulatory transcription factor 1 (MTF1) signaling axis, leading to enhanced expression of MMP-13, ADAMTS-5, and other catabolic enzymes [31]. Activation of this pathway also promotes the expression of hypoxia-inducible factor-2α (HIF-2α), recognized as one of the major regulators of catabolic processes in OA [32,37]. The ZIP8–MTF1 axis is currently regarded as one of the key molecular mechanisms driving cartilage degradation and disease progression in osteoarthritis [31,32].

Overall, current evidence indicates that disturbances in zinc homeostasis actively contribute to OA development by promoting extracellular matrix degradation and enhancing catabolic signaling pathways [3,31,32].

Importantly, a recent meta-analysis did not identify a significant difference in serum zinc concentrations between patients with OA and healthy controls, indicating that the relationship between circulating zinc levels and OA is not straightforward [35] (Table 1).

### 3.2. Copper

Copper plays a crucial role in maintaining connective tissue integrity and protecting joint cells against oxidative stress [3,8,22]. It serves as a cofactor for copper/zinc superoxide dismutase (Cu/Zn-SOD), which neutralizes reactive oxygen species, and for lysyl oxidase, an enzyme responsible for collagen and elastin cross-linking, thereby ensuring the mechanical properties of cartilage and other connective tissues [3,22,38].

Copper also contributes indirectly to iron homeostasis through the activity of ceruloplasmin, a ferroxidase that catalyzes the oxidation of Fe^2+^ to Fe^3+^, facilitating iron binding to transferrin and its subsequent transport in plasma [22].

In recent years, increasing attention has been paid to the potential role of cuproptosis—a copper-dependent form of regulated cell death—in the pathogenesis of OA [39,40]. Disturbances in copper metabolism may lead to mitochondrial dysfunction, enhanced oxidative stress, and chondrocyte injury [38,39,40]. Although this mechanism remains under intensive investigation, it is considered a promising novel component of OA pathogenesis [39,40].

Available evidence suggests that proper copper homeostasis is essential for maintaining redox balance and normal cartilage metabolism [3,22,38].

A recent meta-analysis demonstrated significantly higher serum copper levels in patients with OA than in healthy controls. Moreover, Mendelian randomization analysis suggested a potential causal association between genetically predicted higher serum copper levels and OA risk [35] (Table 1). Clinical evidence also suggests an association between circulating copper and inflammatory activity in OA. In a study of older adults with knee OA, plasma Cu levels were positively associated with both C-reactive protein (CRP; *p* = 0.033) and interleukin-6 (IL-6; *p* = 0.001), and these associations remained significant after adjustment for potential confounding factors, including age, sex, body mass index (BMI), physical activity, smoking, disease severity, total knee arthroplasty, and dietary copper intake [30]. These findings further support a relationship between altered copper homeostasis and systemic inflammatory activity in OA.

### 3.3. Iron

Iron is indispensable for fundamental biological processes, including DNA synthesis, cellular respiration, and mitochondrial function [13,20]. However, excessive iron accumulation exerts potent pro-oxidant effects, leading to cellular damage [13,20,41].

Growing evidence indicates that ferroptosis—an iron-dependent form of regulated cell death—plays an important role in the pathogenesis of OA [41,42,43,44]. A key regulator of this process is glutathione peroxidase 4 (GPX4), which utilizes glutathione to detoxify lipid hydroperoxides [41,42]. Reduced GPX4 activity promotes lipid peroxidation, loss of membrane integrity, and increased susceptibility of chondrocytes to injury [41,42,43,44].

The pathogenic role of iron is also related to its participation in the Fenton reaction, resulting in excessive production of reactive oxygen species [20,41]. Elevated ROS levels promote extracellular matrix degradation, mitochondrial dysfunction, and activation of inflammatory and catabolic signaling pathways [41,42,43,44].

Collectively, current findings suggest that excessive iron accumulation may contribute to OA progression through the induction of ferroptosis and enhancement of oxidative stress [41,42,43,44].

### 3.4. Magnesium

Magnesium participates in several hundred enzymatic reactions and plays an important role in regulating inflammatory responses [3,45]. It exerts anti-inflammatory effects by suppressing activation of the NF-κB signaling pathway and reducing the production of pro-inflammatory cytokines, including IL-1β and TNF-α [45,46].

Moreover, magnesium contributes to cartilage metabolism and bone mineralization by influencing osteoblast and osteoclast activity as well as the biomechanical properties of subchondral bone [3,45,46]. Experimental studies further suggest that magnesium deficiency enhances chondrocyte apoptosis, increases inflammatory mediator production, and disrupts cartilage remodeling [45,46].

Current evidence indicates that adequate magnesium status may contribute to the maintenance of joint homeostasis through its effects on inflammatory regulation, cartilage metabolism, and bone remodeling [3,45,46].

### 3.5. Selenium

Selenium is an essential trace element with an important role in maintaining cellular redox homeostasis and protecting articular cartilage against oxidative damage. Its biological effects are largely mediated through selenoproteins, including glutathione peroxidase 1 (GPX1) and glutathione peroxidase 4 (GPX4), which contribute to the detoxification of reactive oxygen species (ROS) and lipid hydroperoxides. GPX4 is particularly important because it limits membrane lipid peroxidation and thereby protects cells against ferroptotic cell death [41,47,48,49].

Selenium deficiency may impair antioxidant defense by disrupting selenium metabolism and reducing the synthesis of stress-related selenoproteins. Importantly, selenophosphate synthetase 1 (SEPHS1), a regulator of selenium metabolism, has been shown to be downregulated in human and murine osteoarthritic cartilage. Reduced SEPHS1 expression limits the synthesis of selenoproteins with oxidoreductase functions, increases ROS accumulation, and promotes chondrocyte senescence, thereby accelerating cartilage matrix degeneration. Experimental studies further demonstrated that selenium deficiency and SEPHS1 loss may act synergistically to exacerbate OA pathogenesis [35].

Selenium may also influence signaling pathways involved in oxidative stress and inflammation in OA. Experimental evidence indicates that selenium can activate the Nrf2 pathway, promote glutathione synthesis, and increase the expression of antioxidant enzymes, thereby reducing excessive ROS production in chondrocytes. In parallel, selenium-mediated downregulation of NF-κB signaling has been associated with reduced expression of pro-inflammatory cytokines and matrix-degrading proteases. These mechanisms provide a more direct molecular link between selenium status, oxidative stress, inflammation, chondrocyte dysfunction, and cartilage degradation in OA. However, further clinical studies are required to determine whether selenium supplementation provides meaningful therapeutic benefits in patients with OA [48].

Consistent with these mechanistic observations, a recent meta-analysis reported significantly lower serum selenium levels in patients with OA than in healthy controls [35] (Table 1).

### 3.6. Manganese

Manganese functions as a cofactor for mitochondrial superoxide dismutase (MnSOD), one of the principal antioxidant enzymes protecting cells against oxidative stress. MnSOD neutralizes reactive oxygen species generated within mitochondria, thereby protecting chondrocytes from oxidative damage [50,51].

In addition to its antioxidant role, manganese participates in proteoglycan synthesis and cartilage metabolism, contributing to the biosynthesis of key extracellular matrix components [3,50]. Manganese deficiency may reduce MnSOD activity, promote mitochondrial dysfunction, and impair cartilage repair processes [50,51].

Clinical evidence is consistent with a potential disturbance of manganese homeostasis in OA, as a recent meta-analysis demonstrated significantly lower serum manganese levels in patients with OA than in healthy controls [35] (Table 1).

### 3.7. Summary

In summary, available evidence indicates that essential metals and trace elements are important not only for maintaining the physiological function of articular cartilage and subchondral bone but may also participate in molecular mechanisms relevant to OA pathogenesis [3,8,31,41]. Disturbances in their homeostasis may influence matrix metalloproteinase activity, oxidative stress, regulated cell death pathways—including ferroptosis and cuproptosis—inflammatory responses, mitochondrial function, and extracellular matrix remodeling [31,39,41,45,47,50]. Importantly, the effects of individual elements are context-dependent, and both deficiency and excessive accumulation may disrupt joint homeostasis through distinct molecular mechanisms. Although alterations in circulating concentrations of selected elements have been reported in patients with OA, their diagnostic, prognostic, and therapeutic relevance remains to be established in well-designed clinical studies.

## 4. Toxic Metals and Environmental Exposure

Unlike essential trace elements, cadmium (Cd), lead (Pb), mercury (Hg), and arsenic (As) have no established essential physiological role in humans and are considered here primarily in the context of environmental exposure. Their inclusion in this review is therefore not intended to provide a general overview of metal toxicity, but to critically evaluate whether environmental exposure to these elements may contribute to OA-related processes. Importantly, the strength of evidence differs considerably among individual toxic metals, and direct evidence linking some of these elements to OA remains limited [11,12,52].

### 4.1. Cadmium

Among toxic metals, cadmium has attracted particular attention because several mechanisms of cadmium toxicity overlap with pathways involved in OA pathogenesis. Cadmium exposure promotes excessive generation of reactive oxygen species (ROS), impairs antioxidant defense, and disrupts mitochondrial function, thereby creating a pro-oxidative cellular environment [52,53,54]. These effects are particularly relevant to articular cartilage, where persistent oxidative stress can impair chondrocyte homeostasis and promote degenerative processes [55,56].

Cadmium may additionally activate inflammatory signaling pathways, including NF-κB and MAPK, resulting in increased expression of pro-inflammatory mediators such as IL-1β, IL-6, and TNF-α [53,54]. Because these pathways also regulate cartilage catabolism and matrix-degrading enzymes in OA, cadmium-induced oxidative and inflammatory signaling provides a plausible mechanistic link between environmental exposure and cartilage damage. Experimental evidence further indicates that cadmium can promote chondrocyte injury and cell death, thereby reducing the population of viable cells responsible for maintaining and repairing the extracellular matrix [55,56].

Nevertheless, although these mechanistic observations support the biological plausibility of a contribution of cadmium to OA-related tissue damage, the available evidence does not establish cadmium exposure as an independent causal factor for OA. Further studies directly evaluating exposure levels, metal accumulation in joint tissues, and structural or clinical OA outcomes are therefore required.

### 4.2. Lead

Lead differs from cadmium in that its potential relevance to OA is closely related to its strong affinity for mineralized tissues. Bone represents a major reservoir of accumulated lead, which may persist for prolonged periods and interfere with physiological bone remodeling [52,57]. Lead exposure has been associated with alterations in osteoblast and osteoclast function, impaired mineralization, and changes in bone properties [57,58,59,60].

These effects may be relevant to OA because subchondral bone is an active component of the osteochondral unit and undergoes substantial remodeling during disease progression. Disturbances in osteoblast and osteoclast activity may alter subchondral bone architecture and biomechanical properties, potentially influencing the mechanical and biochemical environment of the overlying articular cartilage. Thus, the potential contribution of lead to OA should be considered primarily in the context of chronic skeletal accumulation and disruption of bone–cartilage homeostasis rather than solely as a consequence of general systemic toxicity [57,58,59,60].

However, the relationship between lead exposure and OA remains incompletely defined, and mechanistic findings concerning bone toxicity should not be interpreted as direct evidence of OA causation. Additional longitudinal and tissue-specific studies are needed to determine whether chronic lead exposure contributes independently to OA initiation or progression.

### 4.3. Mercury and Arsenic: Potential Mechanisms and Current Evidence Gaps

In contrast to cadmium and lead, direct evidence linking mercury exposure to OA remains limited. Mercury is a potent immunotoxic agent capable of affecting immune-cell function, cytokine production, and immune tolerance [61,62]. Alterations in the balance between Th17 and regulatory T (Treg) cells are relevant to autoimmune responses [63]. However, these mechanisms have been investigated predominantly in the context of systemic toxicity and autoimmune disorders rather than OA. Therefore, although mercury-induced immune dysregulation may be relevant to rheumatic inflammation more broadly, its specific contribution to OA pathogenesis remains uncertain [61,62,63].

Arsenic exposure is associated with mitochondrial dysfunction, oxidative stress, and disturbances in cellular bioenergetics [64,65]. Experimental studies have also demonstrated interactions between arsenic exposure and NOD-like receptor family pyrin domain-containing 3 (NLRP3) inflammasome signaling; however, the direction and magnitude of these effects appear to depend on the experimental model and exposure conditions [66,67]. These mechanisms overlap with pathways implicated in OA, but current evidence does not establish that arsenic-induced mitochondrial or inflammasome alterations directly drive OA pathology.

Accordingly, mercury and arsenic are included here primarily as environmental toxicants with biological effects that intersect with molecular pathways relevant to rheumatic diseases. Their inclusion should not be interpreted as evidence that either metal is an established causal factor for OA. OA-specific experimental, epidemiological, and joint-tissue studies are needed before their contribution to disease initiation or progression can be defined more precisely.

### 4.4. Summary

Collectively, toxic metals may influence musculoskeletal tissues through several mechanisms that overlap with established pathways of OA, including oxidative stress, mitochondrial dysfunction, inflammatory signaling, immune dysregulation, chondrocyte injury, and disturbed bone remodeling [52,53,54,55,56,57,58,59,60,61,62,63,64,65]. However, the available evidence is heterogeneous and differs substantially among individual metals. Cadmium and lead currently have clearer mechanistic relevance to cartilage or bone homeostasis, whereas the OA-specific evidence for mercury and arsenic remains comparatively limited. Toxic metals should therefore be considered potential environmental modifiers of OA-related biological processes rather than established primary causes of the disease. Further longitudinal studies integrating quantitative exposure assessment with metal measurements in joint tissues and well-defined OA outcomes are required to clarify these relationships.

## 5. Trace Elements in Other Rheumatic Diseases: A Comparative Perspective

Although OA represents the primary focus of this review, disturbances in trace element homeostasis have also been reported in other rheumatic diseases. A brief comparison with rheumatoid arthritis (RA), systemic lupus erythematosus (SLE), ankylosing spondylitis (AS), and gout is included solely to provide a comparative context for the OA-focused discussion and to distinguish OA-specific evidence from broader alterations in trace element homeostasis across rheumatic disorders. Although these diseases differ substantially in their underlying pathophysiology, alterations in metal and trace element metabolism may intersect with common processes such as oxidative stress, immune regulation, inflammation, and bone remodeling [4,7,8,15,24,68].

### 5.1. Rheumatoid Arthritis

Among inflammatory rheumatic diseases, disturbances in trace element metabolism are particularly well described in RA. Alterations in circulating zinc and copper concentrations have been reported in patients with RA and may be associated with systemic inflammation and disease activity [5,6,24]. Zinc participates in the regulation of lymphocyte, macrophage, and antigen-presenting cell function, whereas increased circulating copper may partly reflect enhanced ceruloplasmin synthesis during the inflammatory response [15,24,69,70].

Iron metabolism is also markedly affected by chronic inflammation. IL-6-induced hepcidin production reduces circulating iron availability and promotes iron sequestration within macrophages, contributing to anemia of chronic disease and alterations in redox homeostasis [13,17,23]. Selenium status may additionally influence antioxidant capacity through selenoproteins such as glutathione peroxidase [71]. Thus, in RA, altered trace element concentrations may reflect both systemic inflammatory activity and disturbances in redox and immune homeostasis [5,6,24,71]. Clinical evidence further supports an association between trace element status and inflammatory activity in RA. In a study of 102 patients with RA, circulating copper levels were positively correlated with CRP (r = 0.45), ESR (r = 0.58), and DAS28-CRP (r = 0.35), whereas circulating iron levels showed inverse correlations with CRP (r = −0.46), erythrocyte sedimentation rate (ESR; r = −0.55), and Disease Activity Score 28 based on C-reactive protein (DAS28-CRP; r = −0.37) (all *p* < 0.01) [24]. These findings suggest that alterations in circulating Cu and Fe may partly reflect systemic inflammatory burden and disease activity rather than representing disease-specific abnormalities.

### 5.2. Other Rheumatic Diseases

Alterations in trace element concentrations have also been reported in SLE, including changes in zinc, copper, iron, and selenium [7]. However, their mechanistic contribution to disease-specific pathways, such as type I interferon signaling and neutrophil extracellular trap (NET) formation, remains incompletely defined [72,73,74,75]. Current evidence therefore supports an association between altered trace element status, oxidative stress, and chronic immune activation, but does not establish trace element dyshomeostasis as a primary driver of SLE pathogenesis.

In AS, chronic inflammation coexists with profound alterations in bone remodeling, including pathological new bone formation [76,77]. The IL-23/IL-17 axis plays an important role in inflammatory signaling and skeletal changes associated with spondyloarthritis [78,79]. Alterations in trace element status have also been reported in AS; however, their mechanistic contribution to disease-specific inflammatory and bone-remodeling pathways remains insufficiently established. Therefore, trace element abnormalities may be considered potential modifiers or biomarkers of inflammatory and metabolic disturbances rather than established drivers of AS pathogenesis.

In gout, monosodium urate crystal deposition and subsequent activation of the NLRP3 inflammasome remain the principal drivers of acute inflammation [80,81,82]. Oxidative stress, mitochondrial dysfunction, and ionic disturbances can modulate inflammasome activity; however, the specific contribution of individual trace elements to gout pathogenesis has not yet been clearly established. Thus, although metal-dependent processes may intersect with inflammasome regulation, current evidence is insufficient to define a direct metal-dependent mechanism in gout. A comparative overview of the principal trace element alterations, proposed mechanisms, and strength of current evidence across OA and other rheumatic diseases is presented in Table 2.

### 5.3. Summary

Overall, disturbances in trace element homeostasis occur across several rheumatic diseases, but their biological significance and the strength of supporting evidence vary considerably among disease entities. The available evidence is strongest for selected alterations in RA, whereas the mechanistic roles of trace elements in SLE, AS, and gout remain less clearly defined. These observations provide a useful comparative context for OA but should not be interpreted as evidence of a uniform metal-dependent mechanism across rheumatic diseases.

## 6. Molecular Mechanisms Linking Metal and Trace Element Dyshomeostasis to Osteoarthritis

Metal and trace element dyshomeostasis may contribute to OA pathogenesis through several interconnected molecular mechanisms, including oxidative stress, ferroptosis, mitochondrial dysfunction, dysregulation of intracellular signaling pathways, activation of the NLRP3 inflammasome, and increased activity of enzymes responsible for extracellular matrix degradation [2,4,41,42,43,44,51,80,81,82,91]. These mechanisms do not operate independently but form an interconnected network in which redox disturbances, metabolic dysfunction, inflammatory signaling, regulated cell death, and cartilage catabolism may mutually reinforce one another during OA progression [2,4,91].

### 6.1. Oxidative Stress

One of the fundamental molecular mechanisms linking metal dyshomeostasis to OA is oxidative stress, resulting from an imbalance between the generation of reactive oxygen species and the cellular capacity to neutralize them. The most important mediators include reactive oxygen species (ROS), such as superoxide anion, hydrogen peroxide, and hydroxyl radical, as well as reactive nitrogen species (RNS), including nitric oxide and peroxynitrite. Their excessive production leads to oxidative damage to lipids, proteins, and nucleic acids and activates inflammatory and catabolic signaling pathways [2,4,91].

Metals and trace elements can influence redox homeostasis through several complementary mechanisms. Redox-active metals, particularly iron and copper, may promote ROS generation when present in excess, whereas selenium, zinc, and manganese contribute to antioxidant defense through trace element-dependent antioxidant systems [3,4,47,48,49,50,91]. Selenium is required for the activity of selenoproteins such as glutathione peroxidase 1 (GPX1) and glutathione peroxidase 4 (GPX4), while manganese acts as a cofactor for mitochondrial MnSOD [47,48,49,50,51]. Consequently, both excessive accumulation of redox-active metals and impaired availability of essential trace elements may shift the redox balance of chondrocytes toward oxidative stress.

In OA chondrocytes, excessive ROS production has consequences that extend beyond direct molecular damage. Oxidative stress can impair mitochondrial function, which may further enhance ROS generation and establish a self-amplifying cycle of cellular injury [2,4,91]. ROS also modulate redox-sensitive transcription factors, particularly NF-κB and activator protein 1 (AP-1), promoting the expression of pro-inflammatory mediators and catabolic enzymes involved in extracellular matrix degradation [2,91]. Through these mechanisms, oxidative stress shifts chondrocyte metabolism from maintenance of extracellular matrix homeostasis toward a more inflammatory and catabolic phenotype.

Thus, oxidative stress represents an important mechanistic link between disturbed metal homeostasis and OA progression. Rather than acting as an isolated consequence of metal imbalance, redox dysregulation interacts with mitochondrial dysfunction, inflammatory signaling, regulated cell death, and extracellular matrix degradation, which are discussed in greater detail in the following sections [2,4,91].

### 6.2. Ferroptosis

Ferroptosis is an iron-dependent form of regulated cell death characterized by excessive accumulation of lipid peroxides and failure of cellular antioxidant defense mechanisms. In OA, ferroptosis has emerged as an important mechanism linking disturbed iron homeostasis, oxidative stress, chondrocyte dysfunction, and extracellular matrix degradation [41,42,43,44].

Iron overload increases the intracellular pool of ferrous iron (Fe^2+^), which promotes ROS generation through the Fenton reaction. Excessive ROS enhance the peroxidation of polyunsaturated fatty acids within cellular membranes, resulting in the accumulation of toxic lipid hydroperoxides and progressive membrane damage. Acyl-CoA synthetase long-chain family member 4 (ACSL4) contributes to this process by promoting the incorporation of polyunsaturated fatty acids into membrane phospholipids, thereby increasing their susceptibility to lipid peroxidation. Consequently, increased intracellular Fe^2+^ availability together with enhanced lipid peroxidation creates a cellular environment favoring ferroptotic death [42,43].

A major protective mechanism against ferroptosis is the system Xc^−^–glutathione (GSH)–glutathione peroxidase 4 (GPX4) axis. SLC7A11, a functional component of the cystine/glutamate antiporter system Xc^−^, facilitates cystine uptake required for GSH synthesis. GSH subsequently serves as an essential cofactor for GPX4, which reduces lipid hydroperoxides and prevents their accumulation in cellular membranes. Therefore, suppression of SLC7A11, depletion of GSH, or reduced GPX4 expression or activity impairs antioxidant defense and increases chondrocyte susceptibility to ferroptosis [41,42,43].

Importantly, ferroptosis-related alterations have been demonstrated in experimental models of OA. Inflammatory stimulation and iron overload are associated with increased ROS production and lipid peroxidation together with alterations in ferroptosis-regulating proteins, including GPX4, SLC7A11, p53, and ACSL4. Experimental inhibition of ferroptosis has been shown to reduce ferroptotic features and chondrocyte damage, further supporting the involvement of this form of regulated cell death in OA progression [41,42,43,44].

Ferroptosis is also closely interconnected with mitochondrial dysfunction and cartilage catabolism. Iron accumulation, oxidative stress, and lipid peroxidation may impair mitochondrial function, while mitochondrial dysfunction can further enhance oxidative injury. Ferroptotic chondrocyte damage may additionally disturb extracellular matrix homeostasis and promote a more catabolic cartilage phenotype. Thus, ferroptosis provides an important mechanistic link between iron dyshomeostasis, oxidative stress, chondrocyte death, and progressive cartilage degeneration in OA [41,42,43,44].

### 6.3. Mitochondrial Dysfunction

Mitochondria play a central role in cellular energy metabolism, redox homeostasis, calcium signaling, and the regulation of cell survival. In OA chondrocytes, mitochondrial dysfunction is characterized by impaired oxidative phosphorylation, reduced adenosine triphosphate (ATP) production, increased mitochondrial reactive oxygen species (mtROS) generation, loss of mitochondrial membrane potential, and disturbances in mitochondrial dynamics and quality control. These alterations compromise chondrocyte homeostasis and promote oxidative stress, inflammatory responses, cellular senescence, and extracellular matrix degradation [83,84].

Mitochondrial dysfunction and oxidative stress form a self-amplifying cycle in OA. Impairment of the mitochondrial respiratory chain increases electron leakage and mtROS production, whereas excessive ROS further damage mitochondrial proteins, membrane lipids, and mitochondrial DNA (mtDNA). The accumulation of mitochondrial damage consequently reduces bioenergetic efficiency and further increases ROS generation. In chondrocytes, this vicious cycle promotes cellular dysfunction and may activate inflammatory and cell-death pathways, thereby contributing to progressive cartilage degeneration [83].

Mitochondrial dysfunction in OA is also closely associated with disturbances in Ca^2+^ homeostasis. Mitochondrial Ca^2+^ uptake is tightly controlled by the mitochondrial calcium uniporter (MCU) complex and its regulatory components. Endoplasmic reticulum (ER)–mitochondria contact sites also contribute to mitochondrial Ca^2+^ homeostasis. Recent evidence from temporomandibular joint OA demonstrated that dysregulation of the inositol 1,4,5-trisphosphate receptor (IP3R)–glucose-regulated protein 75 (GRP75)–voltage-dependent anion channel 1 (VDAC1) alters ER-to-mitochondria Ca^2+^ transfer, promoting mitochondrial Ca^2+^ overload and chondrocyte apoptosis [36]. Recent experimental evidence has demonstrated that disruption of mitochondrial calcium uptake 1 (MICU1)-dependent regulation of mitochondrial Ca^2+^ influx in chondrocytes promotes pathological mitochondrial Ca^2+^ accumulation, excessive ROS generation, bioenergetic dysfunction, and apoptosis, supporting a direct link between disturbed mitochondrial Ca^2+^ handling and chondrocyte injury [51,90].

Importantly, mitochondrial dysfunction should not be regarded solely as a downstream consequence of oxidative stress. Metal dyshomeostasis may directly compromise mitochondrial function through disturbances in redox balance, respiratory chain activity, and ionic homeostasis. Iron overload and ferroptosis can enhance mitochondrial oxidative injury, whereas toxic metals such as cadmium, arsenic, and lead have been associated with mitochondrial toxicity and impaired respiratory function [41,42,43,44,53,54,64,65]. These interactions indicate that metal-induced mitochondrial injury can arise through multiple, mutually reinforcing mechanisms involving redox imbalance, disturbed Ca^2+^ signaling, and regulated cell-death pathways.

Damaged mitochondria may also contribute to inflammation through the release of mitochondrial damage-associated molecular patterns (DAMPs), including mtDNA. These signals can activate innate immune and inflammatory pathways, thereby linking mitochondrial injury with persistent inflammation in the OA joint. Mitochondrial ROS may additionally promote activation of the NLRP3 inflammasome, providing a mechanistic connection between mitochondrial dysfunction, oxidative stress, and the production of pro-inflammatory mediators [80,81,82,83].

Mitophagy, the selective removal of damaged mitochondria, is an essential component of mitochondrial quality control. Under physiological conditions, mitophagy prevents the accumulation of dysfunctional mitochondria and limits excessive mtROS production. In OA, impaired mitophagy promotes the persistence of damaged mitochondria, leading to further ROS accumulation, metabolic dysfunction, inflammatory signaling, chondrocyte senescence or death, and extracellular matrix degradation [83,84]. Conversely, experimental enhancement of mitophagy has been associated with improved mitochondrial homeostasis and attenuation of chondrocyte injury, suggesting that restoration of mitochondrial quality control may represent a potential therapeutic strategy in OA [83,84].

Collectively, mitochondrial dysfunction represents an important convergence point linking metal dyshomeostasis with oxidative stress, disturbed Ca^2+^ homeostasis, ferroptosis, inflammatory signaling, and impaired mitochondrial quality control. The reciprocal interactions among these processes may establish self-amplifying cycles of mitochondrial and cellular injury, ultimately promoting chondrocyte dysfunction, cell death, and progressive cartilage degeneration in OA [41,42,43,44,51,83,84,90].

### 6.4. Signaling Pathways

Intracellular signaling pathways constitute an important link between metal dyshomeostasis, oxidative stress, inflammation, and cartilage degradation. Among the pathways involved in OA pathogenesis, NF-κB, mitogen-activated protein kinase (MAPK), Nrf2, and HIF-2α are particularly relevant to the mechanisms discussed in this review, whereas JAK/STAT and phosphoinositide 3-kinase/protein kinase B (PI3K/Akt) contribute more broadly to inflammatory responses, cell survival, and metabolic regulation in joint tissues [1,32,37,85,91].

NF-κB is one of the major pro-inflammatory and catabolic signaling pathways involved in OA. In chondrocytes, its activation can be induced by pro-inflammatory cytokines, particularly IL-1β and TNF-α, as well as by oxidative stress. Activation of NF-κB signaling promotes nuclear translocation of NF-κB transcription factors and induces the expression of inflammatory mediators and cartilage-degrading enzymes, including MMPs and ADAMTS. This promotes extracellular matrix degradation and can further amplify inflammatory and catabolic signaling within the joint [1,32].

Metal dyshomeostasis may intersect with these catabolic pathways at several levels, with zinc providing one of the best-characterized examples. ZIP8 expression is increased in OA chondrocytes, resulting in enhanced Zn^2+^ influx and activation of the metal-regulatory transcription factor MTF1. MTF1 subsequently promotes the expression of matrix-degrading enzymes, including MMP-3, MMP-13, and ADAMTS-5. The ZIP8–Zn^2+^–MTF1 axis therefore provides a direct molecular link between disturbed zinc homeostasis and cartilage catabolism in OA [31,32].

MAPK signaling provides an additional link between inflammatory and oxidative stimuli and cartilage degradation. Activation of MAPK signaling in chondrocytes contributes to inflammatory and catabolic responses and promotes the expression of matrix-degrading enzymes, thereby facilitating extracellular matrix breakdown and progressive cartilage damage [1,32].

Other signaling networks, including JAK/STAT and PI3K/Akt, also participate in the regulation of inflammatory responses, cell survival, and metabolic activity in joint tissues [32,85]. Crosstalk among these pathways and NF-κB/MAPK signaling further illustrates that OA-associated signaling operates as an interconnected molecular network rather than as a series of independent pathways.

In contrast to predominantly pro-inflammatory and catabolic signaling, Nrf2 serves a protective role by activating antioxidant and cytoprotective genes. Impairment of Nrf2-dependent responses reduces the capacity of chondrocytes to neutralize ROS and may thereby promote oxidative damage and inflammatory signaling. Conversely, Nrf2 activation enhances endogenous antioxidant defenses and can counteract NF-κB-dependent inflammatory responses. This mechanism is particularly relevant to selenium: experimental evidence indicates that selenium can activate Nrf2-dependent antioxidant responses, increase glutathione-related antioxidant capacity, and suppress NF-κB-associated inflammatory signaling in articular chondrocytes [48].

HIF-2α represents an additional catabolic regulator in OA and contributes to the expression of matrix-degrading enzymes. Importantly, reciprocal activation between HIF-2α and the ZIP8–Zn^2+^–MTF1 axis has been demonstrated in OA chondrocytes. HIF-2α upregulates ZIP8, thereby increasing Zn^2+^ influx and MTF1 activity, while the ZIP8–Zn^2+^–MTF1 axis can in turn enhance HIF-2α expression. This positive feedback loop amplifies the expression of matrix-degrading enzymes and promotes cartilage destruction [37].

Collectively, these findings indicate that disturbances in metal and trace element homeostasis can influence OA progression not only through oxidative or toxic effects but also through modulation of interconnected intracellular signaling networks. In particular, the ZIP8–Zn^2+^–MTF1/HIF-2α axis provides a direct link between zinc dyshomeostasis and cartilage catabolism, whereas selenium-dependent modulation of Nrf2 illustrates how trace elements may influence protective antioxidant signaling. The balance between these catabolic and protective mechanisms may ultimately contribute to chondrocyte dysfunction, extracellular matrix degradation, and OA progression.

### 6.5. The NLRP3 Inflammasome

Activation of the NLRP3 inflammasome represents an important molecular mechanism linking oxidative stress, mitochondrial dysfunction, and inflammatory responses in OA. The NLRP3 inflammasome is a multiprotein complex that promotes activation of caspase-1, resulting in the maturation and release of the pro-inflammatory cytokines IL-1β and IL-18. Activated caspase-1 also cleaves gasdermin D (GSDMD), leading to membrane pore formation and pyroptotic cell death. Thus, NLRP3 activation provides a mechanistic link between cellular stress, inflammation, and chondrocyte injury [80,81,82,87].

Several danger signals associated with OA can promote NLRP3 inflammasome activation, including excessive ROS production, mitochondrial dysfunction, release of mitochondrial damage-associated signals, ionic disturbances, and crystal-induced cellular stress. Mitochondrial ROS are particularly important because damaged mitochondria can amplify oxidative stress and facilitate inflammasome activation, thereby connecting mitochondrial dysfunction with persistent inflammatory signaling in the OA joint [80,81,82,83,84].

The ROS/thioredoxin-interacting protein (TXNIP)/NLRP3 axis represents an important redox-sensitive mechanism involved in this process. Under oxidative conditions, TXNIP can promote NLRP3 inflammasome activation, thereby linking disturbances in cellular redox homeostasis with inflammatory signaling. Subsequent activation of caspase-1 promotes the maturation of IL-1β and IL-18 and GSDMD-dependent pyroptosis, amplifying inflammatory responses and chondrocyte damage. Experimental evidence from an in vivo OA model supports the involvement of the TXNIP/NLRP3 pathway in cartilage degeneration and pyroptotic cell injury [87].

Metal dyshomeostasis may contribute to NLRP3 activation primarily through its effects on redox and mitochondrial homeostasis. Iron overload represents a particularly relevant example, as increased intracellular iron availability promotes ROS generation and oxidative injury, creating conditions that favor NLRP3 inflammasome activation. Experimental evidence indicates that iron overload can enhance NLRP3-associated inflammatory responses in chondrocytes and contribute to cartilage degeneration. Other disturbances in metal homeostasis may potentially influence inflammasome activity indirectly through oxidative stress and mitochondrial injury; however, direct metal-specific evidence in OA remains more limited [41,42,43,44,52,53,54,80,81,82,87].

Collectively, activation of the NLRP3 inflammasome may establish a self-amplifying inflammatory cycle in which oxidative and mitochondrial damage promotes inflammasome activation, while the resulting release of IL-1β and IL-18 and pyroptotic cell injury further intensifies local inflammation and cartilage catabolism. Therefore, the NLRP3 inflammasome represents an important mechanistic bridge connecting redox disturbances, mitochondrial dysfunction, inflammation, and progressive joint tissue damage in OA [80,81,82,83,87].

### 6.6. Matrix Metalloproteinases

Progressive degradation of the extracellular matrix (ECM) is a hallmark of OA and represents a common downstream consequence of oxidative stress, inflammatory signaling, mitochondrial dysfunction, and altered metal homeostasis. Matrix metalloproteinases (MMPs) and members of the a disintegrin and metalloproteinase with thrombospondin motifs (ADAMTS) family are among the major proteolytic enzymes responsible for cartilage matrix degradation. In particular, MMP-1, MMP-3, and MMP-13, together with the aggrecanases ADAMTS-4 and ADAMTS-5, play important roles in the progressive loss of cartilage integrity [31,32,88,89].

MMP-13 is particularly important in OA because of its ability to degrade type II collagen, the major collagenous component of articular cartilage. MMP-1 also contributes to collagen degradation, whereas MMP-3 participates in extracellular matrix breakdown and can contribute to the activation of other matrix-degrading enzymes. In parallel, ADAMTS-4 and ADAMTS-5 primarily mediate aggrecan degradation. The combined loss of aggrecan and type II collagen progressively compromises the structural and biomechanical properties of articular cartilage [88,89].

Expression and activity of these proteolytic enzymes are regulated by pro-inflammatory cytokines, oxidative stress, and intracellular signaling pathways, including NF-κB, MAPK, and HIF-2α. In OA chondrocytes, activation of these catabolic pathways shifts cartilage metabolism from matrix maintenance toward matrix degradation, increasing the expression of MMPs and ADAMTS enzymes and thereby accelerating cartilage destruction [32,37,85,88,89,91].

The activity of matrix-degrading enzymes is additionally controlled by endogenous tissue inhibitors of metalloproteinases (TIMPs). Under physiological conditions, the balance between metalloproteinases and their inhibitors contributes to the maintenance of extracellular matrix homeostasis. In OA, disruption of this balance favors proteolytic activity and cartilage catabolism. In particular, insufficient inhibition of MMP and ADAMTS activity may facilitate persistent degradation of collagen and aggrecan [89].

Importantly, zinc is linked to MMP-mediated cartilage degradation at both catalytic and regulatory levels. As zinc-dependent proteolytic enzymes, MMPs require Zn^2+^ for their catalytic activity [88,89]. In addition, altered intracellular zinc homeostasis can regulate the expression of matrix-degrading enzymes. In OA chondrocytes, increased ZIP8-mediated Zn^2+^ influx activates MTF1, which enhances the expression of MMP-3, MMP-13, and ADAMTS-5. Thus, the ZIP8–Zn^2+^–MTF1 axis provides a direct mechanistic link between disturbed zinc homeostasis and extracellular matrix degradation in OA [31]. Other disturbances in metal and trace element homeostasis may contribute more indirectly to matrix degradation by promoting oxidative stress, mitochondrial dysfunction, or pro-inflammatory signaling, which can enhance catabolic responses in chondrocytes.

**Figure 1:**
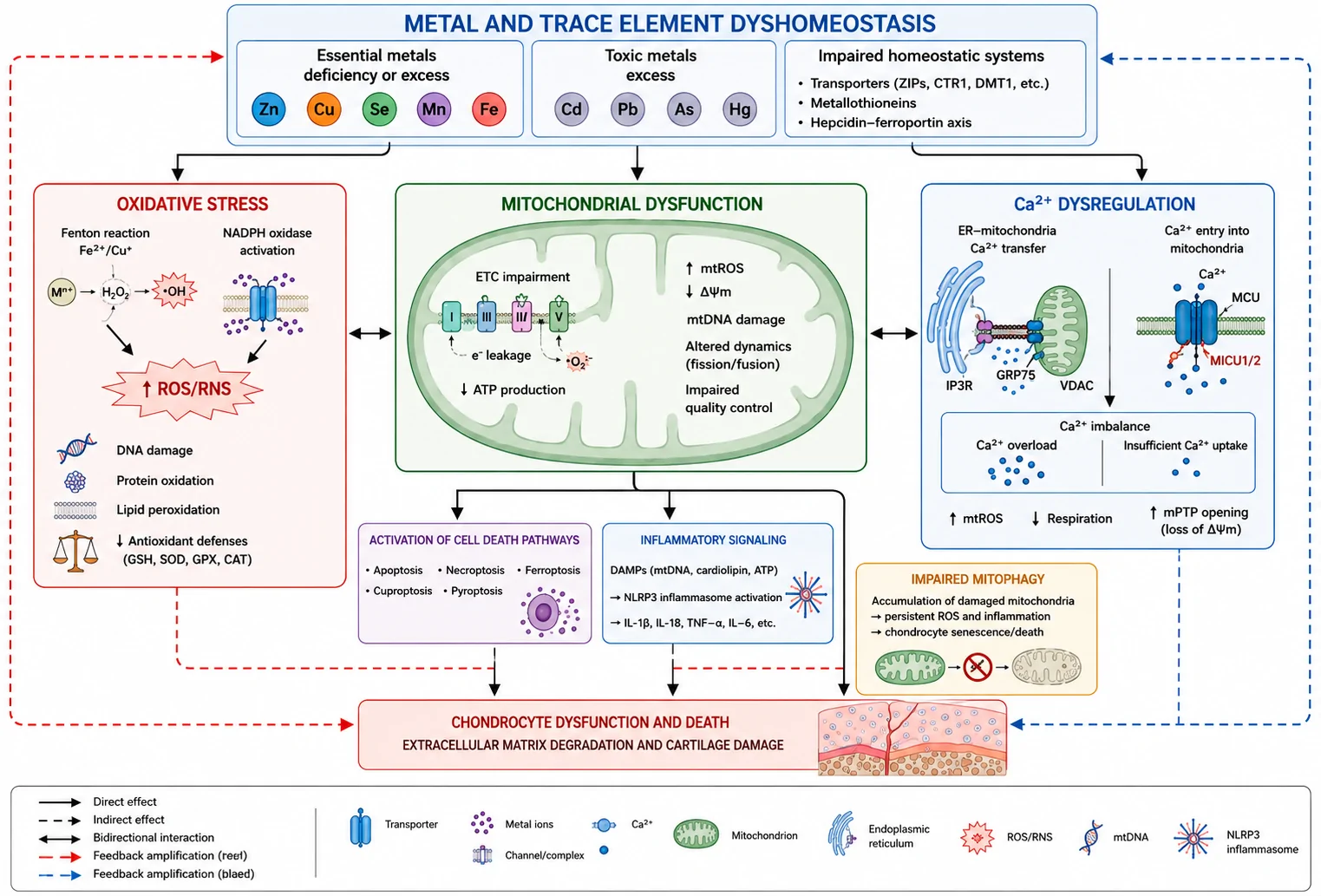
Proposed molecular mechanisms linking metal and trace element dyshomeostasis with the development and progression of osteoarthritis. Disturbances in essential metal and trace element homeostasis, accumulation of redox-active metals, and exposure to toxic metals may promote oxidative stress, mitochondrial dysfunction, Ca^2+^ dysregulation, regulated cell death, and activation of inflammatory and stress-related signaling pathways. Mitochondrial dysfunction may arise both from oxidative stress and from direct metal-induced disturbances in mitochondrial function and ionic homeostasis, while reciprocal interactions among oxidative stress, mitochondrial dysfunction, and Ca^2+^ imbalance may further amplify cellular injury. These interconnected mechanisms contribute to increased production of pro-inflammatory mediators and matrix-degrading enzymes, chondrocyte dysfunction and death, extracellular matrix degradation, and progressive cartilage damage. Abbreviations: ADAMTS, a disintegrin and metalloproteinase with thrombospondin motifs; ECM, extracellular matrix; ER, endoplasmic reticulum; GPX4, glutathione peroxidase 4; MCU, mitochondrial calcium uniporter; MMPs, matrix metalloproteinases; NLRP3, NOD-like receptor family pyrin domain-containing 3; ROS, reactive oxygen species; RNS, reactive nitrogen species.

Collectively, increased MMP and ADAMTS activity represents a major downstream mechanism through which inflammatory, oxidative, metabolic, and metal-dependent disturbances converge to promote cartilage destruction. The resulting degradation of collagen and aggrecan ultimately contributes to progressive loss of cartilage structure and function in OA [31,32,88,89]. The major molecular mechanisms linking metal dyshomeostasis to oxidative stress, inflammation, mitochondrial dysfunction, ferroptosis, and extracellular matrix degradation in OA are summarized in Figure 1.

### 6.7. Summary

In summary, metal and trace element dyshomeostasis may contribute to OA pathogenesis through a network of interconnected molecular mechanisms rather than through a single pathway. Disturbances in redox balance, iron-dependent ferroptosis, mitochondrial dysfunction, dysregulation of intracellular signaling pathways, NLRP3 inflammasome activation, and increased activity of matrix-degrading enzymes collectively contribute to chondrocyte dysfunction, inflammatory and catabolic responses, and progressive extracellular matrix degradation [2,31,32,37,41,42,43,44,48,80,81,82,83,84,85,86,87,88,89,91].

Importantly, these mechanisms interact at multiple levels. Oxidative stress can promote mitochondrial damage and inflammatory signaling, while dysfunctional mitochondria further increase ROS generation and may facilitate NLRP3 inflammasome activation. Iron dyshomeostasis can promote lipid peroxidation and ferroptotic chondrocyte death, whereas altered zinc homeostasis can enhance cartilage catabolism through the ZIP8–Zn^2+^–MTF1/HIF-2α axis. Conversely, selenium-dependent antioxidant mechanisms, including Nrf2-related responses, may counteract oxidative and inflammatory injury. These interconnected processes ultimately converge on increased MMP and ADAMTS activity, loss of collagen and aggrecan, and progressive cartilage degeneration [31,37,41,42,43,44,48,83,84,87,88,89].

Collectively, these findings support the concept that metal and trace element homeostasis is integrated with redox regulation, cellular metabolism, inflammatory signaling, regulated cell death, and extracellular matrix turnover in OA. A better understanding of these interactions may help identify molecular pathways that could be explored as potential therapeutic targets; however, further experimental and clinical studies are required to establish their translational relevance.

## 7. Diagnostic Significance of Metal Homeostasis in Osteoarthritis and Other Rheumatic Diseases

The assessment of metal homeostasis and proteins involved in metal transport and storage has attracted increasing interest as a potential diagnostic and prognostic tool in osteoarthritis and other rheumatic diseases [92,93,94,95]. The development of highly sensitive analytical methods has enabled more accurate determination of metal concentrations, assessment of their chemical forms, and analysis of their distribution in biological material, which has substantially increased interest in using these parameters as biomarkers of rheumatic diseases [93,94,95,96]. This applies both to direct measurements of trace element concentrations, such as zinc (Zn), copper (Cu), iron (Fe), and selenium (Se), and to indirect indicators, including ferritin, hepcidin, ceruloplasmin, and the Cu/Zn ratio. These parameters may reflect inflammatory activity, oxidative stress intensity, immune dysregulation, and the extent of joint tissue damage [17,22,23,92,94].

### 7.1. Biomarkers of Metal Concentrations and Transport Proteins

In rheumatic diseases, changes in serum concentrations of selected metals have been shown to have diagnostic value and may support the assessment of disease activity. In rheumatoid arthritis, the most frequently observed pattern is decreased zinc concentration accompanied by increased copper concentration, with these changes correlating with the intensity of inflammation, C-reactive protein (CRP) levels, erythrocyte sedimentation rate (ESR), and acute-phase markers. Zinc, as an element involved in regulating the function of lymphocytes, macrophages, and antigen-presenting cells, as well as in antioxidant protection, may reflect both nutritional status and the level of chronic immune activation. In contrast, increased copper concentration is partly associated with enhanced synthesis of ceruloplasmin, an acute-phase protein [5,6,24,92].

Measurements of iron, ferritin, hepcidin, and ceruloplasmin are also considered important. In chronic inflammatory diseases, reduced serum iron concentration may coexist with increased ferritin and hepcidin levels, reflecting iron redistribution and activation of the inflammatory response rather than an actual depletion of body iron stores. Ceruloplasmin may serve as an additional indicator of inflammatory activity and disturbances in copper metabolism [17,22,23,94].

Increasing attention is also being paid to selenium measurement, as selenium deficiency may indicate weakened antioxidant protection and greater susceptibility to oxidative damage. Due to its role in selenoprotein function, selenium concentration may serve as a complementary marker of oxidative activity [47,71].

The Cu/Zn ratio appears to be a particularly interesting indicator, as it is considered a marker of the balance between inflammatory processes and the antioxidant capacity of the organism. An increased Cu/Zn ratio has been associated with more pronounced inflammation, higher CRP and ESR values, and a less favorable metabolic and immunological profile. In many studies, this ratio has shown greater diagnostic value than separate measurements of either element alone. In rheumatology, the Cu/Zn ratio may serve as a useful auxiliary biomarker for monitoring disease activity, although it still requires validation in large prospective studies [92,95].

### 7.2. Metals in Synovial Fluid

An important direction in diagnostic research is the analysis of metals in synovial fluid, which reflects the local biochemical environment of the affected joint more accurately than serum. It has been shown that concentrations of zinc, copper, iron, and other elements in synovial fluid may differ from their levels in peripheral blood and may be associated with local inflammatory activity, cartilage degradation, and proteolytic enzyme activity [96,97].

In OA and RA, synovial fluid is increasingly being analyzed as a source of diagnostic and prognostic biomarkers that may help differentiate types of joint inflammation, assess disease severity, and predict progression. Measurement of trace elements in this material may in the future complement the assessment of other inflammatory and degradative biomarkers, particularly when integrated with the analysis of cytokines, extracellular matrix degradation products, and markers of oxidative stress [96,97,98,99].

Growing interest has also focused on exosomes present in synovial fluid. These vesicles transport proteins, lipids, microRNAs, and other signaling molecules, thereby reflecting local pathological processes occurring within the joint. Integrating metal composition analysis with exosome characterization may in the future improve the sensitivity and specificity of diagnostics in rheumatic diseases [98,99].

### 7.3. Metabolomics and Metallomics

Multidimensional approaches, such as metabolomics and metallomics, are gaining increasing importance in the diagnosis of rheumatic diseases. Metabolomics enables the global analysis of changes in biological metabolite profiles, whereas metallomics focuses not only on the quantitative determination of metals but also on the analysis of their chemical forms (speciation), bioavailability, and interactions with proteins and other cellular components [100,101,102,103].

The development of modern analytical techniques, such as inductively coupled plasma mass spectrometry (ICP-MS), laser ablation ICP-MS (LA-ICP-MS), and high-resolution mass spectrometry, enables precise quantification of even trace amounts of elements and assessment of their distribution in tissues and body fluids [101,103].

The combination of metabolomics and metallomics may provide a more comprehensive picture of metabolic, inflammatory, and oxidative disturbances occurring in the course of OA, RA, and other rheumatic diseases. These methods create opportunities to identify new biomarker panels useful not only for disease diagnosis but also for phenotype differentiation, activity assessment, treatment monitoring, and prediction of progression [93,100,101,102,103].

In the future, integration of data on metal concentrations, their mutual ratios, chemical speciation, redox status, and metabolomic changes in serum, urine, and synovial fluid may be of particular importance. Such an approach is consistent with the development of precision medicine and may contribute to more accurate characterization of rheumatic diseases and individualized therapy.

### 7.4. Summary

In summary, metals and proteins involved in their transport and storage represent a promising group of diagnostic and prognostic biomarkers in rheumatic diseases [92,93,94,95,96,97,98,99,100,101,102,103]. Measurements of zinc, copper, iron, and selenium concentrations, parameters such as ferritin, hepcidin, and ceruloplasmin, as well as the Cu/Zn ratio, may reflect inflammatory activity, oxidative stress, and immune dysregulation. Supplementing these data with synovial fluid analysis, exosome assessment, and metabolomic and metallomic tools may substantially improve the precision of diagnosis and disease monitoring in the future. However, it should be emphasized that most biomarkers based on metal analysis have not yet been incorporated into routine diagnostic recommendations of the European Alliance of Associations for Rheumatology (EULAR) or the American College of Rheumatology (ACR), highlighting the need for further validation studies and standardization of analytical methods [96,97,98,99,100,101,102,103].

## 8. Potential Therapeutic Applications

The growing recognition of the role of metals in the pathogenesis of osteoarthritis and other rheumatic diseases has led to their increasing consideration not only as risk factors or biomarkers, but also as potential targets for therapeutic intervention [3,8,41,42,43,44]. Contemporary therapeutic strategies increasingly focus not only on symptom relief but also on modulation of the molecular mechanisms responsible for disease development, which is consistent with the concept of precision medicine [41,42,43,44,86]. These strategies include trace element supplementation, metal chelation, antioxidant therapies, and more targeted molecular approaches, such as modulation of metal transporters and ferroptosis-related pathways [31,41,42,43,44,47,48,86,91]. However, it should be emphasized that most of these approaches remain at the stage of preclinical studies or early translational analyses, and their clinical value requires further confirmation [41,42,43,44,91,104].

### 8.1. Trace Element Supplementation

One of the most intuitive therapeutic approaches is trace element supplementation, particularly in cases where deficiency may exacerbate oxidative stress, immune dysregulation, and joint tissue degradation. Selenium, magnesium, and zinc are among the most frequently studied elements in this context [47,48,70,71,104,105]. Supplementation should, however, be considered cautiously and preferably preceded by laboratory assessment of deficiency, because both deficiency and excess of selected elements may disrupt cellular homeostasis [3,8,70,105].

Selenium has attracted interest due to the role of selenoproteins, particularly glutathione peroxidase, in neutralizing reactive oxygen species. Theoretically, selenium supplementation could enhance antioxidant protection, reduce chondrocyte damage, and modulate chronic inflammation [47,48,71]. The potential significance of selenium has been considered in both OA and RA; however, available clinical data do not yet allow its supplementation to be clearly defined as a standard component of treatment [71].

Magnesium exhibits potential anti-inflammatory effects and supports cartilage and bone metabolism; therefore, adequate magnesium status may have a protective role in joint diseases [3,45,46]. The therapeutic hypothesis assumes that correction of magnesium deficiency could reduce inflammation, improve bone cell function, and favorably influence subchondral bone remodeling [45,46]. In the context of bone metabolism, interactions among magnesium, calcium, and vitamin D should also be considered, as these systems jointly influence bone mineralization and bone cell function.

Similarly, zinc, as an element involved in immune regulation and the activity of numerous enzymes, may exert protective effects [70,104,105]. At the same time, its role is complex, because excessive activation of the ZIP8–MTF1 axis may promote catabolic processes in cartilage [31]. Therefore, potential zinc supplementation should be considered in close relation to the actual status of zinc metabolism and with attention to the risk of disrupting its local homeostasis in joint tissues [31,70,105].

### 8.2. Metal Chelators

Another potential therapeutic approach involves metal chelators, compounds capable of binding metal ions and limiting their toxic effects. This strategy appears particularly relevant for iron and toxic metals, whose excess may enhance oxidative stress, mitochondrial damage, and cell death [41,42,43,44,52,53,54]. In the context of OA and other rheumatic diseases, chelation could theoretically reduce Fe^2+^-dependent redox reactions, limit tissue damage, and inhibit certain mechanisms of ferroptosis [41,42,43,44,106].

The best-characterized iron chelators include deferoxamine (DFO), deferasirox (DFX), and deferiprone (DFP). In experimental studies, deferoxamine has attracted particular interest because pharmacological iron chelation reduced iron accumulation in articular cartilage and attenuated OA-associated cartilage lesions, supporting the potential chondroprotective effects of controlled iron reduction [106]. These findings suggest that selective reduction in pathologically increased iron availability may represent a potential therapeutic strategy aimed at cartilage protection [41,42,43,44,106].

Despite promising biological rationale, the use of metal chelators in rheumatology remains limited. A major concern is the possibility of disrupting the physiological homeostasis of essential elements and the risk of adverse effects resulting from excessive reduction in their bioavailability [41,42,43,44,106]. Therefore, this approach requires high selectivity and precise identification of patients in whom excess of a given metal plays a meaningful pathogenic role.

### 8.3. Antioxidant Therapies

Because oxidative stress represents one of the central mechanisms of tissue damage in rheumatic diseases, antioxidant therapies constitute a natural direction of investigation. Their aim is to reduce excessive ROS and RNS production, protect mitochondria, and decrease activation of inflammatory and catabolic pathways [2,4,91]. In practice, this may involve both supplementation with trace elements supporting antioxidant systems and the use of compounds with direct antioxidant activity [47,48,91,107,108,109,110].

The potential efficacy of such interventions is based on their ability to interrupt the vicious cycle linking oxidative stress, NF-κB activation, pro-inflammatory cytokine production, and increased metalloproteinase activity [2,91]. Experimental and clinical studies have investigated compounds such as N-acetylcysteine (NAC), curcumin, resveratrol, and melatonin [107,108,109,110]. NAC, as a precursor of glutathione, may support the cellular capacity to neutralize oxidative stress [107]. Curcumin and resveratrol exert anti-inflammatory and antioxidant effects, partly through modulation of the NF-κB pathway [108,109]. Melatonin may support mitochondrial homeostasis and reduce chondrocyte ferroptosis [110].

However, translating promising experimental findings into clear clinical benefit remains challenging. The greatest potential may lie not in nonspecific “scavenging” of free radicals, but rather in more precise restoration of redox balance, mitochondrial protection, and activation of endogenous cytoprotective mechanisms such as the Nrf2 pathway [86,106,110].

### 8.4. Targeting Metal Transporters and Ferroptosis

A particularly interesting and modern therapeutic direction involves targeting metal transporters that regulate cellular metal influx, distribution, and efflux. In OA, the zinc transporter ZIP8 (SLC39A8) has received the greatest attention and is one of the best-characterized zinc transporters in the pathogenesis of this disease [31]. Its overexpression in chondrocytes activates the ZIP8–MTF1 axis and increases the expression of cartilage-degrading enzymes such as MMP-13 and ADAMTS-5 [31]. Inhibition of this pathway could theoretically reduce catabolic processes and slow the progression of degenerative joint changes [31,32].

Zinc transporter (ZnT; SLC30) family members may also have therapeutic relevance, as they are responsible for zinc efflux from the cytoplasm or its sequestration into cellular organelles. Modulation of ZIP and ZnT transporter activity could in the future enable more precise control of intracellular zinc homeostasis and thereby influence inflammatory pathways, degradative enzymes, and cell survival [31,70,105]. At present, however, these remain mainly experimental concepts, since excessive inhibition of zinc transport could impair its physiological functions.

The therapeutic modulation of ferroptosis is also increasingly linked to metal transport and iron metabolism [41,42,43,44]. Because ferroptosis depends on Fe^2+^ availability, lipid peroxidation, and failure of protective mechanisms such as GPX4, its modulation may represent a promising therapeutic target [41,42,43,44,111]. In OA studies, ferroptosis inhibitors such as ferrostatin-1 and liproxstatin-1 have been shown to reduce ferroptotic features in chondrocytes, while deferoxamine may additionally decrease iron availability and activate antioxidant responses [106,111]. These strategies remain at the preclinical stage and require further evaluation of safety and efficacy.

The main characteristics of essential trace elements and toxic metals involved in osteoarthritis and other rheumatic diseases are summarized in Table 3.

### 8.5. Summary

In summary, potential therapeutic applications related to the role of metals in rheumatic diseases include supplementation with selected trace elements, reduction in toxic metal effects through chelation, antioxidant therapies, and more selective strategies targeting metal transporters and ferroptosis mechanisms [31,41,42,43,44,47,48,70,91,104,105,106,107,108,109,110,111]. The development of therapies directed at metal homeostasis is consistent with the concept of precision medicine, in which treatment selection is based on an individual patient’s molecular and biochemical profile. Although most of the approaches discussed remain at the stage of preclinical or early clinical investigation, their further development may contribute to more effective treatments for osteoarthritis and other rheumatic diseases.

## 9. Limitations of Current Research and Future Directions

Despite growing interest in the role of metals in the pathogenesis of osteoarthritis and other rheumatic diseases, the current state of knowledge remains affected by numerous methodological and interpretative limitations. These primarily include study heterogeneity, small patient cohorts, lack of standardization in analytical measurements, and difficulties in assessing cause-and-effect relationships. Such limitations hinder the comparison of results, the formulation of definitive conclusions, and the translation of experimental observations into clinical practice [93,94,100,101,102,103,112].

### 9.1. Study Heterogeneity

One of the major challenges is the considerable heterogeneity of available studies. This applies to the characteristics of the studied populations, the type of biological material analyzed, laboratory methods, and selected endpoints. Individual studies have assessed metal concentrations in serum, plasma, whole blood, urine, synovial fluid, or tissue samples, which makes direct comparison of results and data synthesis difficult [93,94,101].

An additional source of heterogeneity arises from clinical differences between patients, including age, sex, disease stage, inflammatory activity, coexisting metabolic diseases, dietary patterns, and treatment regimens. As a result, findings obtained in one patient group cannot always be easily generalized to other populations [112]. This problem is particularly relevant in studies on OA, which itself is a heterogeneous disease in terms of clinical phenotype and underlying biological.

### 9.2. Small Patient Cohorts

Another limitation is the small size of study populations, which is characteristic of many studies assessing metal concentrations and biomarkers related to metal metabolism. Small sample sizes reduce statistical power, increase the risk of random error, and make it difficult to detect subtle but biologically meaningful associations. This problem is especially relevant for single-center studies and investigations involving less commonly measured elements or more specialized analytical techniques [113].

Small patient cohorts also limit the ability to perform reliable subgroup analyses, for example according to disease stage, inflammatory activity, type of treatment, or presence of comorbidities. Consequently, many observations remain preliminary and require confirmation in larger, multicenter studies [113].

### 9.3. Lack of Standardization of Measurements

A major obstacle to interpreting results is the lack of standardization of analytical measurements. Different studies use different laboratory techniques, sample preparation procedures, storage conditions for biological material, and reporting methods [101,102,103,114]. This applies both to the measurement of metals themselves and to proteins involved in their transport and storage, such as ferritin and ceruloplasmin [17,22,23].

The lack of standardization makes it difficult to establish reference values, determine clinically relevant cut-off points, and compare results between centers. In the case of synovial fluid biomarkers, the problem is even greater because sample composition is additionally influenced by local inflammatory conditions, sampling technique, possible sample dilution, and coexisting tissue damage. Similar challenges apply to metabolomics and metallomics studies, where the absence of unified analytical and bioinformatic procedures remains a significant limitation [100,101,102,103,114].

### 9.4. Difficulties in Assessing Causality

One of the most important limitations is the difficulty in determining whether observed disturbances in metal homeostasis are a cause, a consequence, or merely an accompanying marker of the disease process. Most available studies are observational and cross-sectional, meaning that they can demonstrate the coexistence of certain changes but do not allow definitive assessment of cause-and-effect relationships [112,115].

For example, decreased zinc or selenium levels may contribute to enhanced oxidative stress and inflammatory responses, but they may also be secondary consequences of chronic inflammation, redistribution of elements within the body, or disease-associated nutritional changes [5,47,71]. Similarly, increased copper and ferritin levels may result from activation of the acute-phase response rather than reflecting a primary role of these factors in disease initiation. Assessment of causality is further complicated by the effects of treatment, supplementation, comorbidities, and environmental factors [17,22,23].

Therefore, further prospective, multicenter, and mechanistic studies are needed to better distinguish primary from secondary phenomena and to determine which alterations in metal homeostasis have true pathogenic and therapeutic significance [115].

### 9.5. Summary

In summary, current research on the role of metals in rheumatic diseases provides many promising observations; however, interpretation is limited by substantial methodological heterogeneity, small patient cohorts, lack of standardization in analytical measurements, and difficulties in assessing causality. Overcoming these challenges will be essential for the further development of reliable biomarkers and effective therapeutic strategies based on disturbances in metal homeostasis [93,100,101,102,103,112,113,114,115,116].

## 10. Future Research Directions

Further research into the role of metals in the pathogenesis of osteoarthritis and other rheumatic diseases will likely move toward increasingly integrated and high-resolution biological analytical methods [93,94,101,102,103,117]. Current evidence indicates that measuring the concentration of individual elements in serum or synovial fluid alone is insufficient to fully understand their biological significance [28,96,117]. Future studies should therefore integrate data on metal homeostasis with information on gene expression, cellular metabolic status, spatial distribution of tissue alterations, and individual clinical characteristics of patients [93,94,117,118,119,120,121,122,123,124].

### 10.1. Metallomics

One of the most important future directions is metallomics, which enables not only quantitative determination of metals but also the analysis of their chemical species, interactions with proteins, and distribution across different biological compartments. This approach may facilitate more accurate identification of which metal species play a true pathogenic role and which merely represent secondary markers of ongoing disease processes [101,102,103,117].

High-spatial-resolution techniques, such as laser ablation inductively coupled plasma mass spectrometry (LA-ICP-MS), may be particularly valuable because they enable visualization of metal distribution within tissues [101,103,117]. In the future, metallomics may play a key role in identifying more specific disease biomarkers, characterizing inflammatory and degenerative phenotypes, and monitoring treatment response [93,101,102,103,117]. Combining metallomic analyses with investigations of synovial fluid, synovial membrane, and articular cartilage appears especially promising, as it may provide more precise information than serum measurements alone [28,96,117].

### 10.2. Single-Cell Analysis

Another promising research direction is the development of technologies capable of evaluating biological alterations at the level of individual cells. In rheumatic diseases, single-cell RNA sequencing (scRNA-seq) has emerged as a particularly valuable tool for identifying heterogeneous cellular populations and characterizing their transcriptional profiles. This approach may facilitate the identification of distinct subpopulations of chondrocytes, synovial fibroblasts, macrophages, and other cells involved in inflammation and tissue degradation [118,119]. Consequently, it may become possible to determine which cell populations are most susceptible to disturbances in metal homeostasis.

Particular attention should be paid to the expression of genes encoding metal transporters, antioxidant proteins, ferroptosis-related molecules, and inflammatory mediators. Such analyses may clarify how disturbances in zinc, iron, selenium, or copper homeostasis influence the function of specific cell populations within affected joints and other diseased tissues [31,41,42,43,44,118,119].

### 10.3. Spatial Tissue Analysis

Single-cell analyses can be complemented by methods that preserve information about the spatial localization of molecular alterations within tissues. This is particularly important in joint diseases because pathological processes do not occur uniformly but rather involve specific regions of articular cartilage, synovial membrane, subchondral bone, and tendon insertion sites [120,121]. Such approaches may provide precise information on where metal-dependent pathways, oxidative stress, and inflammatory processes are activated.

One particularly promising technology is spatial transcriptomics, which enables simultaneous analysis of gene expression while preserving spatial tissue architecture [120,121]. This approach may facilitate correlation of local gene expression changes with the presence of specific metals or disturbances in their transport [101,117,120,121]. Such information may substantially improve our understanding of the spatial organization of degenerative and inflammatory changes and help identify tissue regions that are most responsive to targeted therapies [120,121].

### 10.4. Multi-Omics Approaches

Another highly promising direction is the development of multi-omics strategies integrating genomics, transcriptomics, proteomics, metabolomics, and metallomics [93,94,122,123]. Because rheumatic diseases are multifactorial disorders involving numerous overlapping biological pathways, analyses focusing on only one level of regulation are often insufficient [93,94,122]. Integrating multiple layers of biological information may provide a more comprehensive understanding of the complex relationships among metal exposure, redox balance, immune cell activation, cartilage degradation, and bone remodeling [93,94,122,123].

Multi-omics approaches may be particularly valuable for identifying novel disease phenotypes and distinguishing patient subgroups characterized by different dominant pathogenic mechanisms [93,94,122,123]. However, integration of such large-scale biological datasets will require advanced artificial intelligence (AI) and machine learning techniques capable of identifying complex molecular patterns and constructing predictive models. These technologies may ultimately enable more accurate selection of diagnostic and therapeutic strategies tailored to an individual patient’s biological profile [122,123,124].

### 10.5. Personalized Medicine

A natural consequence of these technological advances is the development of personalized medicine. In the future, assessment of an individual’s metal homeostasis, inflammatory pathway activity, susceptibility to oxidative stress, and molecular biomarker profile may allow more accurate prediction of disease progression and more precise therapeutic decision-making [93,124,125,126].

In clinical practice, this could enable identification of patients most likely to benefit from supplementation with specific trace elements, antioxidant therapies, ferroptosis-targeted interventions, or modulation of metal transporters [31,41,42,43,44,47,48,106,124]. Instead of applying a uniform therapeutic strategy to all patients, treatment could gradually be tailored according to each patient’s biological phenotype [124,125,126]. In the longer term, the development of digital twin models may enable simulation of disease progression and prediction of therapeutic response in individual patients [125].

### 10.6. Novel Biomarkers and Targeted Therapies

One of the major objectives of future research is the identification of novel biomarkers that are more sensitive and specific than simple measurements of serum metal concentrations [93,96,126]. These may include composite indices based on elemental ratios as well as integrated panels incorporating metallomic, transcriptomic, metabolomic, and inflammatory biomarkers [93,94,101,102,103,122,123]. Concepts related to liquid biopsy, particularly the analysis of extracellular vesicles and exosomes in synovial fluid and other biological specimens, are also attracting increasing attention [126]. Such biomarkers could facilitate early disease diagnosis, prediction of disease progression, and monitoring of therapeutic efficacy [93,96,126].

At the same time, targeted therapies directed against specific components of metal-dependent pathways—including ZIP8 and ZnT transporters, ferroptosis regulators, antioxidant response pathways, and extracellular matrix-degrading enzymes—are likely to continue evolving [31,41,42,43,44,86,88,89,106]. Particularly promising are strategies capable of selectively modulating pathological aspects of metal homeostasis without disrupting their essential physiological functions throughout the body [31,41,42,43,44,106].

### 10.7. Summary

In summary, future research on the role of metals in rheumatic diseases will likely focus on integrating biological information across multiple levels of organization, from individual cells to the whole organism [93,94,117,118,119,120,121,122,123,124,125,126]. Advances in metallomics, single-cell technologies, spatial analyses, multi-omics approaches, artificial intelligence, and personalized medicine offer substantial opportunities to improve our understanding of the complex pathogenic mechanisms underlying rheumatic diseases, identify novel biomarkers, and develop more precise targeted therapeutic strategies [117,118,119,120,121,122,123,124,125,126].

## 11. Conclusions

The evidence presented in this review indicates that metals and trace elements constitute an important component of the complex network of molecular mechanisms responsible for the development and progression of osteoarthritis (OA) and other rheumatic diseases [3,8,31,41,42,43,44,86,93]. Their role extends far beyond basic metabolic functions, as they participate in the regulation of oxidative stress, immune responses, cartilage metabolism, bone remodeling, and the activity of enzymes responsible for extracellular matrix degradation [2,3,4,31,41,42,43,44,86,91]. Among the elements with the greatest biological and pathogenic significance are zinc, iron, copper, magnesium, and selenium, whereas exposure to toxic metals such as cadmium, lead, mercury, and arsenic may further promote inflammatory and degenerative processes [31,41,42,43,44,45,46,47,48,49,50,51,52,53,54,70,71,72,73,74,75,76,77,78,79,80,81,82,127].

One of the most important conclusions emerging from current research is the dual role of metals in rheumatic diseases [3,8,31,41,42,43,44]. Both deficiency and excess of these elements may disrupt the homeostasis of joints and other tissues affected by the disease process [17,22,23,41,42,43,44]. Deficiency of essential trace elements may impair the activity of antioxidant enzymes, disrupt immune cell function, and compromise cartilage and bone metabolism [47,48,70,71,127]. Conversely, excess accumulation of certain metals, particularly iron and toxic metals, may enhance reactive oxygen species production, mitochondrial dysfunction, ferroptosis, and activation of inflammatory signaling pathways [41,42,43,44,52,53,54,86,91].

Particular attention should be paid to zinc, which is involved both in the regulation of immune function and in the activation of cartilage-degrading enzymes through the ZIP8–MTF1 signaling axis [31,32]. Iron also plays a crucial role through its involvement in the Fenton reaction and ferroptosis [41,42,43,44], while copper contributes to antioxidant defense and collagen maturation [37,38]. Magnesium and selenium appear to be particularly important for their anti-inflammatory and antioxidant properties, as well as for maintaining normal metabolism of joint tissues [45,46,47,48]. At the same time, toxic heavy metals may enhance chronic inflammation, autoimmunity, and damage to osteoarticular structures [49,50,51,52,53,54,72,73,74,75,76,77,78,79,80,81,82], highlighting the importance of environmental exposure as a contributing factor in the pathogenesis of rheumatic diseases.

The accumulated evidence suggests that a better understanding of the mechanisms regulating metal homeostasis may have important clinical implications [93,101,102,103,122,123,124,125,126]. Such knowledge may facilitate the identification of novel biomarkers of disease activity, progression risk, and therapeutic response, while also contributing to the development of more precise therapeutic strategies, including trace element supplementation, modulation of metal transporters, antioxidant therapies, and interventions targeting ferroptosis [93,96,104,105,106,107,108,109,110,111,112,113,114,115,116,117,118,119,120,121,122,123,124,125,126]. In the future, integration of metallomic, transcriptomic, and clinical data, supported by artificial intelligence–based analytical approaches, may provide the foundation for implementing more personalized strategies for the diagnosis and treatment of rheumatic diseases [101,102,103,117,118,119,120,121,122,123,124,125,126].

In conclusion, the role of metals in the pathogenesis of osteoarthritis and other rheumatic diseases is complex, multidimensional, and highly dependent on the biological context [3,86,93]. This complexity highlights that metals should be regarded not merely as passive indicators of metabolic alterations but as active participants in inflammatory, oxidative, degenerative, and reparative processes [31,41,42,43,44,86]. Future investigations employing advanced omics technologies together with precision medicine approaches are expected to substantially expand the diagnostic and therapeutic possibilities available in modern rheumatology [117,118,119,120,121,122,123,124,125,126].

## Figures and Tables

**Table 1:** Representative serum reference intervals and reported alterations of selected essential metals and trace elements in osteoarthritis.

Element	Biological Matrix	Representative Reference Interval in Healthy Adults	Reported Alteration in OA vs. Healthy Controls	Reference
Zinc (Zn)	Serum	800–1625 µg/L	No significant difference; SMD = −0.020 (95% CI: −0.077 to 0.038; *p* = 0.503)	[33,34,35]
Copper (Cu)	Serum	684–1668 µg/L	Higher in OA; SMD = 0.118 (95% CI: 0.061–0.175; *p* < 0.001)	[33,34,35]
Selenium (Se)	Serum	36.8–83.5 µg/L	Lower in OA; SMD = −0.138 (95% CI: −0.209 to −0.068; *p* < 0.001)	[33,34,35]
Manganese (Mn)	Serum	0.622–3.560 µg/L	Lower in OA; SMD = −0.180 (95% CI: −0.326 to −0.034; *p* = 0.016)	[33,34,35]
Iron (Fe)	Serum	546–2177 µg/L	No consistent OA-specific diagnostic threshold established	[33,34]
Magnesium (Mg)	Serum	Male: 0.71–0.93 mmol/L; Female: 0.70–0.91 mmol/L	No consistent OA-specific diagnostic threshold established	[36]

Abbreviations: CI, confidence interval; OA, osteoarthritis; SMD, standardized mean difference. Reference intervals are population-, sex-, matrix-, and method-dependent and should not be interpreted as OA-specific diagnostic thresholds. Reference intervals for Zn, Cu, Se, Mn, and Fe were derived from a healthy adult population and calculated as the 2.5th–97.5th percentiles [33,34]. Sex-specific reference intervals are presented for Mg [36]. Reported alterations in OA for Zn, Cu, Se, and Mn are based on pooled effect estimates from a recent meta-analysis [35].

**Table 2:** Comparative overview of trace element alterations in osteoarthritis and other rheumatic diseases.

Disease	Main Reported Trace Element Alterations	Proposed Biological Relevance	Strength/Limitations of Current Evidence	References
OA	Zn, Cu, Fe, Mg, Se, Mn; exposure to toxic metals	Oxidative stress, mitochondrial dysfunction, ferroptosis/cuproptosis, inflammatory signaling and ECM degradation	Extensive mechanistic evidence supports the involvement of metal-dependent processes; reported concentration changes remain matrix- and population-dependent	[2,25,26,28,29,30,31,32,35,35,37,38,39,40,41,42,43,44,45,46,47,48,49,50,51,83,84,85,86,87,88,89,90]
RA	↓ Zn and altered/increased Cu frequently reported; inflammation-related Fe redistribution; altered Se status	Immune regulation, oxidative stress, IL-6–hepcidin–ferroportin axis and inflammatory activity	Relatively strong clinical evidence, although concentrations vary among studies and biological matrices	[5,6,10,13,17,23,24,28,71]
SLE	Alterations in Zn, Cu, Fe and Se	Potential relationship with oxidative stress and immune dysregulation	Associations reported, but direct links with disease-specific mechanisms remain insufficiently established	[7,72,73,74,75]
AS	Alterations in several trace elements have been reported	Potential interaction with inflammation and bone remodeling	Limited disease-specific mechanistic evidence	[8,76,77,78,79]
Gout	No consistent disease-specific trace element pattern established	Possible intersection with oxidative/mitochondrial stress and NLRP3 regulation	Direct metal-dependent contribution remains insufficiently established	[80,81,82]

Abbreviations: AS, ankylosing spondylitis; ECM, extracellular matrix; IL, interleukin; NLRP3, NOD-like receptor family pyrin domain-containing 3; OA, osteoarthritis; RA, rheumatoid arthritis; SLE, systemic lupus erythematosus.

**Table 3:** Summary of the biological functions, molecular mechanisms, diagnostic significance, and therapeutic implications of essential trace elements and toxic metals involved in osteoarthritis and other rheumatic diseases.

Metal/Trace Element	Main Biological Functions	Major Molecular Mechanisms in Rheumatic Diseases	Diagnostic Significance	Potential Therapeutic Implications
Zinc (Zn)	Immune regulation, enzyme cofactor, cartilage metabolism	ZIP8–MTF1 activation, MMP and ADAMTS expression, immune modulation	Serum Zn, Cu/Zn ratio	Zinc supplementation *, ZIP8 modulation
Iron (Fe)	Oxygen transport, mitochondrial metabolism	ROS generation, Fenton reaction, ferroptosis	Ferritin, hepcidin, serum iron	Iron chelators, ferroptosis inhibitors
Copper (Cu)	Collagen maturation, antioxidant defense	Cu/Zn-SOD activity, cuproptosis, iron homeostasis	Ceruloplasmin, serum Cu	Restoration of copper homeostasis
Selenium (Se)	Antioxidant defense	GPX1/GPX4 activity, inhibition of oxidative stress and ferroptosis	Serum Se	Selenium supplementation *
Magnesium (Mg)	Bone metabolism, anti-inflammatory activity	NF-κB inhibition, regulation of inflammatory cytokines	Serum Mg	Magnesium supplementation *
Manganese (Mn)	Antioxidant defense	MnSOD activity, mitochondrial protection	Experimental biomarker	Correction of deficiency
Cadmium (Cd)	Toxic metal	Oxidative stress, apoptosis, NF-κB activation	Exposure biomarker	Exposure reduction
Lead (Pb)	Toxic metal	Impaired bone remodeling, osteoblast dysfunction	Exposure biomarker	Exposure reduction
Mercury (Hg)	Toxic metal	Immune dysregulation, Th17/Treg imbalance	Exposure biomarker	Exposure reduction
Arsenic (As)	Toxic metal	Mitochondrial dysfunction, NLRP3 inflammasome activation	Exposure biomarker	Exposure reduction

* when deficiency is confirmed. Abbreviations: ADAMTS, a disintegrin and metalloproteinase with thrombospondin motifs; GPX, glutathione peroxidase; MMP, matrix metalloproteinase; MTF1, metal-regulatory transcription factor 1; NF-κB, nuclear factor kappa B; NLRP3, NOD-like receptor family pyrin domain-containing 3; ROS, reactive oxygen species; SOD, superoxide dismutase; Th17, T helper 17; Treg, regulatory T; ZIP8, Zrt- and Irt-like protein 8.

## Data Availability

No new data were created or analyzed in this study. Data sharing is not applicable to this article.
